# Oral mucosal lesions in skin diseased patients attending a dermatologic clinic: a cross-sectional study in Sudan

**DOI:** 10.1186/1472-6831-11-24

**Published:** 2011-09-19

**Authors:** Nada M Suliman, Anne N Åstrøm, Raouf W Ali, Hussein Salman, Anne C Johannessen

**Affiliations:** 1 Section for pathology, The Gade Institute, University of Bergen, Bergen, Norway; 2 Department of Clinical Dentistry, University of Bergen, Bergen, Norway; 3 Faculty of Dentistry, University of Science and Technology, Umdurman, Sudan; 4 Dermatology Clinic, Khartoum Teaching Hospital, Khartoum, Sudan; 5 Haukeland University Hospital, Bergen, Norway

## Abstract

**Background:**

So far there have been no studies focusing on the prevalence of a wide spectrum of oral mucosal lesions (OML) in patients with dermatologic diseases. This is noteworthy as skin lesions are strongly associated with oral lesions and could easily be neglected by dentists. This study aimed to estimate the frequency and socio-behavioural correlates of OML in skin diseased patients attending outpatient's facility of Khartoum Teaching Hospital - Dermatology Clinic, Sudan.

**Methods:**

A cross-sectional hospital-based study was conducted in Khartoum from October 2008 to January 2009. A total of 588 patients (mean age 37.2 ± 16 years, 50.3% females) completed an oral examination and a personal interview of which 544 patients (mean age 37.1 ± 15.9 years, 50% females) with confirmed skin disease diagnosis were included for further analyses. OML were recorded using the World Health Organization criteria (WHO). Biopsy and smear were used as adjuvant techniques for confirmation. Data were analysed using the Statistical Package for Social Science (Version 15.0.1). Cross tabulation and Chi-square with Fisher's exact test were used.

**Results:**

A total of 438 OML were registered in 315 (57.9%, males: 54.6% versus females: 45.6%, p < 0.05) skin diseased patients. Thus, a certain number of patients had more than one type of OML. *Tongue lesions *were the most frequently diagnosed OML (23.3%), followed in descending order by *white lesions *(19.1%), *red and blue lesions *(11%) and *vesiculobullous diseases *(6%). OML in various skin diseases were; *vesiculobullous reaction pattern *(72.2%), *lichenoid reaction pattern *(60.5%), *infectious lesions *(56.5%), *psoriasiform reaction pattern *(56.7%), and *spongiotic reaction pattern *(46.8%). Presence of OML in skin diseased patients was most frequent in older age groups (62.4% older versus 52.7% younger, p < 0.05), in males (63.2% males versus 52.6% females, p < 0.05), patients with a systemic disease (65.2% with systemic versus 51.9% without systemic disease, p < 0.05) and among current users of smokeless tobacco (toombak) (77% current use versus 54.8% no use, p < 0.00).

**Conclusions:**

OML were frequently diagnosed in skin diseased patients and varied systematically with age, gender, systemic condition and use of toombak. The high prevalence of OML emphasizes the importance of routine examination of oral mucosa in a dermatology clinic.

## Background

Epidemiological studies of oral mucosal lesions (OML) are rare globally in comparison with studies on caries and periodontal diseases [1]. Whilst caries and periodontal diseases constitute the most prevalent oral diseases worldwide, cancrum oris, oral manifestations of HIV/AIDS, and oral cancer constitute the main burden of oral diseases in deprived communities in sub Saharan Africa [2]. As the pattern of oral diseases vary across countries, site specific epidemiological studies are needed to address the most commonly occurring oral diseases in order to plan for oral health care service [1,3]

To estimate the prevalence, incidence, distribution and causal factors of OML, studies from the general population are needed. However, population based studies are difficult to carry out because they are expensive and time consuming. The most extensive surveys on OML have been reported from Sweden, America, Malaysia and India [4-7]. Thus, the majority of publications are based upon selected population groups; hospital attendees, the elderly, school children and patients with specific diseases, such as hepatitis C, diabetes, renal and skin diseases [8-16]. Absence of use of standardized methodological design in epidemiological studies of OML has shown substantial disparity in the prevalence of these lesions across different settings worldwide. In general, previous studies have shown that OML tend to increase with age and being a male, and also with lifestyle patterns such as tobacco and alcohol consumption [6,12,17].

In oral medicine, dermatologic diseases have got special attention as OML may be the primary clinical feature or the only sign of various mucocutaneous diseases [18-20]. Focusing on patients referred to a dermatologic clinic, Ramirez-Amador et al [21] reported a prevalence of 35% OML in subjects affected with mucocutaneous conditions. Pemphigus vulgaris, lichen planus, candidiasis, and recurrent aphthous ulcers were the most frequently diagnosed conditions [21]. Yet, there has been no studies focusing on the prevalence of a wide spectrum of different types of OML in patients with dermatologic diseases. This is noteworthy as a certain amount of skin lesions are strongly associated with oral lesions and could be neglected by dentists due to lack of information and/or improper diagnosis [22]. Dentists are often the first to be consulted by patients who develop acute oro-facial pain. Therefore, improving the knowledge about the frequency and diversity of OML at the dermatology clinic will strengthen and enhance interdisciplinary and multispectral approaches as opposed to a single sector approach in the management of such patients. Moreover, OML in skin diseases deserve special attention, considering that some are life-threatening, while others have great impact on individuals and society in terms of pain, discomfort and social and functional limitations [1]. In the Sudan, studies on OML have focused on toombak (Sudanese smokeless tobacco)-associated lesions since several clinical and epidemiological studies have identified toombak use as a possible risk factor for oral cancer [23,24].

### Purpose

The purpose of this study was to estimate the frequency, diversity and socio-behavioural correlates of different types of OML in adult patients with dermatological diseases attending outpatient's facility of Khartoum Teaching Hospital (KTH) - Dermatology Clinic, Sudan.

## Methods

### Sampling procedure

A cross sectional hospital-based study was carried out focusing on patients aged 18 years and above with skin lesions, attending an outpatient dermatologic clinic at KTH from October 2008 to January 2009. KTH is the largest national hospital in Sudan. It is an open public and referral hospital receiving patients from all states of the country. A minimum sample size of 500 patients was calculated based on an assumed prevalence of OML in skin diseased patients of 5%, a confidence interval of 95%, and an absolute precision of 0.02. All patients (n = 4235) attending the outpatient facility during the survey period were invited to participate in the study. The patients were informed in detail about the study procedure and that they could decline at any time without negative consequences, after having given consent.

A total of 1540 subjects (36.4%) accepted verbally to participate in the study. Fear of taking biopsy for asymptomatic lesions and time consuming examinations (oral examination, interview, and biopsy when needed) were the main reasons for not volunteering to participate. Some refusals did not give reason for non-participation. Among those who initially accepted to participate, 544 (544/1540, 35.3%) patients were included in the study. Reasons for none consenting were patients' disappearance and limited resources. Confidentiality of the patients was maintained. The participants were informed about their oral conditions, and health education was provided. Those who needed dental services were referred to the University of Science and Technology (UST), Faculty of Dentistry, for further investigation and management. Participation was voluntary. Written informed consent or finger print for participation and publication of the study was obtained from patients or their parents/guardians. The research conformed to the Helsinki Declaration and ethical clearance, and approval letters were obtained by the participating institutions' committees (UST and KTH, Department of Dermatology, in Sudan). In Norway, the ethical approval was obtained from the Regional Committee for Medical Ethics in Research.

### Interview

A face-to-face interview was conducted by two trained dentists. The structured interview schedule contained questions regarding socio-demographics (gender, age, education, occupation and place of residence during the last 5 years), health and oral health related characteristics and lifestyle (smoking, use of toombak or alcohol). The interview schedule was constructed in English and then translated and used in Arabic. Forward and backward translations were done by two independent Sudanese professional translators in Arabic and English language. Oral health related behaviours were assessed in terms of use of toombak, alcohol and smoking. Use of alcohol and use of toombak was assessed using a 5-point scale: (1) Every day; (2) Several times a week; (3) Sometimes; (4) Never; (5) Former use. Two dummy variables were constructed yielding the categories 0 = never (including the original categories 4), 1 = yes (including the original categories 1, 2, 3 and 5). Smoking habit was assessed using a 4-points scale: (1) Every day; (2) Sometimes; (3) Former use; (4) Never. Those scales were dichotomized into 1 = smoke (including the original categories1, 2 and 3), 0 = never smoke (including the original categories 4).

### Skin examination

An expert dermatologist (HS) evaluated the patient's dermatological disease through information obtained in a structured interview conducted in the outpatient department of the dermatology clinic. Elements evaluated during skin examination were chief complains, and duration and history of chief complains. Past history and family history were also recorded.

### Clinical oral examination

Systematic comprehensive extra-oral and intra-oral clinical examinations based on visual inspection and palpation, following the World Health Organization (WHO) criteria for field surveys [25] were carried out by a dentist (NMS) who received a standard training in diagnosis of OML before the data collection (The Gade Institute, Section for Pathology, and Department of Clinical Dentistry-Section for Oral Surgery and Oral Medicine, University of Bergen, Norway). Oral examination was performed with the subject lying on a medical couch in the outpatient's section of the Department of Dermatology, KTH. All instruments used for oral examination and biopsy were obtained from UST. A head light and an artificial light, mouth mirrors, spatulas, and sterile gauze were used. Occasionally, a cotton swab was used to remove debris to test whether a white lesion could be wiped off. Dentures were removed prior to examination. In those cases requiring further examination; diascopy, smears for *Candida albicans*, punch and incision biopsies were performed to establish precise accurate diagnosis. In addition, selected sections were stained for examination of *Candida albicans *or melanin. Final diagnoses of all lesions were confirmed by an expert oral pathologist (ACJ). Skin lesions and OML encountered during the survey were photographed using a digital camera (Canon EOS 400D).

Clinical parameters were recorded using a structured questionnaire modified from the WHO OML form assessment [25,26]. Parameters which were recorded were; chief complains, disease history, clinical features of the lesion, anatomical location, size, colour, past history, medications used, and associated etiological factors. Self-reported condition of the oral mucosa was also ascertained by asking the patients about dryness of mouth, ulceration, pain, difficulties in swallowing, and burning sensation. The clinical diagnoses of OML were sorted into 14 disease groups, and the total number of types of lesions within each disease group was assessed. In addition, the total number of patients who were diagnosed with any lesion in each separate disease group was counted. Individual patient could have more than one type of OML diagnosed. Consequently, the number of OML would exceed the number of patients.

### Diagnostic criteria for oral mucosal lesions

An OML was defined as any abnormal change or any swelling on the oral mucosal surface. Diagnostic criteria for OML were based on Axell criteria and those defined in previous studies and reviews [5,25,27,28]. Thus, median rhomboid glossitis was defined as asymptomatic, smooth to lobulated well demarcated erythematous zone, surrounded by a sharp furrow that affects midline of posterior dorsal tongue. Atrophy of tongue papillae not compatible with the criteria set for median rhomboid glossitis, has been registered as atrophy of tongue papillae. Vitiligo was defined as depigmented macules and patches that have relatively distinct and possibly hyperpigmented margins present in the lips. The lesion should associate with diagnosed vitiligo elsewhere in the skin. Lichenoid lesions were defined as lesions that have in common basal cell damage, have a lichen planus like aspect, but that lack one or more characteristic clinical aspects [29]. Erythema was defined as redness of the mucosa, caused by hyperemia of the mucosal capillaries. The lesion should disappear on finger pressure (blanching).

In addition to strictly intraoral lesions, angular cheilitis and perioral dermatitis were also recorded. Linea alba, cheek biting, leukoedema, lingual varicose, Fordyce's granules, and excessive melanin racial pigmentation were excluded from the study.

### Statistical analysis

Data were analyzed using the Statistical Packages for Social Sciences (SPSS, version 15.0). The level of statistical significance was set at 5%. Cross tabulation and Chi-square with Fisher's exact test were used to test the statistical significance of the relationships between skin disease groups and types OML on the one hand side and socio-behavioural variables on the other.

## Results

### Sample profile

A total of 544 patients with a skin disease diagnosis participated in the present study. The mean age was 37.1 ± 15.9 years (range 18-85), 50% were females and 77% were permanent residents of Khartoum during the previous 5 years. Males were more frequently employed than females (72.6% versus 27.4%, p < 0.001), whereas use of smoking, toombak or alcohol was more reported in males than females (p < 0.05). Totals of 17.7%, 12.7% and 4.3% confirmed former or current smoking, use of toombak and alcohol use, respectively (Table 1).

**Table 1:** Socio-demographic and behavioural distribution of patients with skin disease by sex (n = 544)

Variables	Female n (%)	Male n (%)	Total n (%)
***Age***			
Younger (18-32 yrs)	144 (51.6)	135 (48.4)	279 (52.2)
Older (33-85 yrs)	123 (48.2)	132 (51.8)	255 (47.8)
***Occupation***			
Employed	89 (27.4)	236 (72.6)*	325 (59.9)
Unemployed	183 (83.9)	35 (16.1)	218 (40.1)
***Education***			
Lower education (illiterate/primary school)	143 (53.6)	124 (46.4)	267 (49.9)
Higher education	126 (47)	142 (53)	268 (50.1)
***Residence***			
Residence last 5 yr: Khartoum	207 (49.9)	208 (50.1)	415 (77)
Residence last 5 yr: outside	65 (52.4)	59 (47.6)	124 (23)
***Medical history***			
No systemic condition	138 (46.5)	159 (53.5)	297 (54.6)
Presence of systemic condition	134 (54.3)	113 (45.7)	247 (45.4)
***Smoking***			
Never	263 (59.5)	179 (40.5)	442 (82.3)
Former/current use	6 (6.3)	89 (93.7)**	95 (17.7)
***Toombak use***			
Never	263 (56.3)	204 (43.7)	467 (87.3)
Former/current use	8 (11.8)	60 (88.2)**	68 (12.7)
***Alcohol use***			
Never	269 (52.5)	243 (47.5)	512 (95.7)
Former/current use	2 (8.7)	21 (91.3)**	23 (4.3)

• * p < 0.05 ** p < 0.001

• The total number in the different categories did not add to 544 owing to missing values

### Skin diseases profile

**Figure 1:**
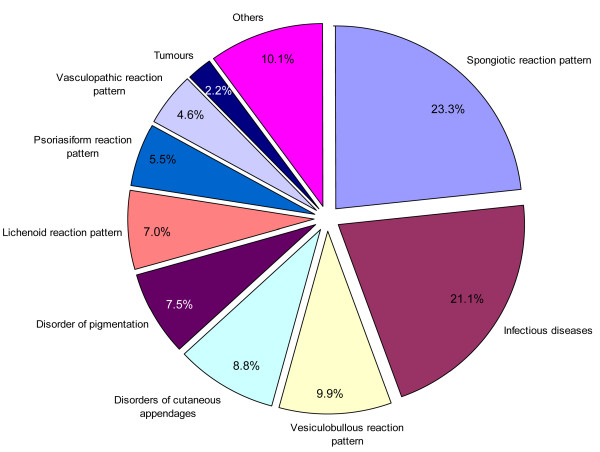
**Frequencies of skin disease categories**.

Ninety-four different types of skin lesions, grouped into 22 categories of skin diseases, were diagnosed. The categories of skin diseases that affected less than 10 patients (13 of the 22 categories) were grouped together and labelled "others". *Spongiotic reaction pattern *was the most frequently diagnosed dermatological disease group (126/544, 23.2%), followed in descending order by *skin infectious diseases *(115/544, 21.1%, i.e. fungal infections 9.6%, viral infection 6.8%, bacterial infection 2.9%, and protozoal infection 1.8%), *vesiculobullous reaction pattern *(54/544, 9.9%), and *disorders of cutaneous appendages *(48/544, 8.8%). The least frequently diagnosed group was *tumours *(12/544, 2.2%) (Figure 1). *Disorder of pigmentation *was more common in females than in males (78% versus 22%, p < 0.001). *Vesiculobullous reaction pattern *and *disorders of cutaneous appendages *were most common in older (32.7% versus 67.3%) and younger (85.4% versus 14.6%) patients, respectively (p < 0.05).

### Oral mucosal lesions profile

In total, 315 of the 544 patients included in the study had at least one clinically recognized type of OML (57.9%). A certain amount of the patients had more than one type of OML, thus the total number of OML recorded in the 315 patients was 438. Of those affected, 202 (64.1%) had one type of OML, 78 (24.8%) had two types of OML, and 35 (11.1%) had three or more types of OML. A total of 51 different clinical diagnoses were recorded. For each patient, one type of OML was only recorded once, although in some patients the OML could be manifested at several locations. Only 15.9% (n = 50) of the patients agreed to undergo punch biopsy confirmation. Absence of epithelial dysplasia was confirmed in all biopsies taken from lesions such as oral leukoplakia, frictional lesion, and snuff dipper's lesions. The age of patients affected by OML ranged from 18 to 81 years, with an average of 38.6 years (±16.5).

As shown in Table 2, *tongue lesions *were the most frequently diagnosed OML (23.3%) followed in descending order by *white lesions *(19.1%), *red and blue lesions *(11%) and *vesiculobullous diseases *(6%). The least frequently diagnosed OML group was *malignant tumours *(0.2%). *White lesions *(42.2% versus 57.8%) and the *red and blue lesions *(37.3% versus 62.7%) occurred most frequently in older patients (p < 0.05). *Ulcerative conditions *were most frequently diagnosed in males (18% versus 6%, p < 0.05). Coated tongue (48.0%) and tongue tie (3.1%) were the most frequently occurring OML within *tongue lesions*, whereas snuff dipper lesions/toombak-associated lesions (28.8%), erythema (48.3%), and pemphigus vulgaris (46.9%) were the most frequently occurring diagnoses in *white lesions, red and blue lesions and vesiculobullous diseases*, respectively. A total of 0.2% of the patients investigated presented with Kaposi's sarcoma, the only lesion in the group of *malignant tumours*.

**Table 2:** Prevalence of oral mucosal lesions in the total group of skin diseased patients (n = 544), in the group of skin diseased patients with OML and within the 14 most frequently occurring OML groups

Oral mucosal lesions	Proportion with a specific lesion in the total group of patients (n = 544)	Proportion with a specific lesion in the total group of patients with any OML (n = 315)	Proportion with specific lesion within 14 OML groups	Biopsy
***Tongue lesions***	***n (%)***	***%***	***%***	
Coated tongue	61 (11.2)	19.4	48.0	
Fissured tongue	26 (4.8)	8.3	20.5	
Geographic tongue	23 (4.2)	7.3	18.1	2(+ve)*
Atrophy of tongue papillae	17 (3.1)	5.4	13.4	1(+ve)
Geographic tongue+Fissured tongue	12 (2.2)	3.8	9.4	
Tongue-tie	4 (0.7)	1.3	3.1	
Total number of patients with any tongue lesion ¶	127 (23.3)	40.3		
***White lesions***				
Snuff dipper's lesion	30 (5.5)	9.5	28.8	8(+ve)
Frictional lesions	25 (4.6)	7.9	24.0	4(+ve)
Leukoplakia	17 (3.1)	5.4	16.3	5(+ve)
Vitiligo	16 (2.9)	5.1	15.4	
Nicotine stomatitis	7 (1.3)	2.2	6.7	
Lichen planus	5 (0.9)	1.6	4.8	3(+ve)
Lupus erythematosus	4 (0.7)	1.3	3.8	1(-ve)§
Unspecified nicotine stomatitis	3 (0.6)	1.0	2.9	
Lichenoid lesions	1 (0.2)	0.3	1.0	1(+ve)
Total number of patients with any white lesion ¶	104 (19.1)	33.0		
***Red and blue lesions***				
Erythema	29 (5.3)	9.2	48.3	2(-ve)
Petechia	25 (4.6)	7.9	41.7	
Erosion	7 (1.3)	2.2	11.7	
Hemangioma	3 (0.6)	1.0	5.0	
Total number of patients with any red and blue lesion ¶	60 (11)	19.0	100	
***Vesiculobullous diseases***				
Pemphigus vulgaris	15(2.8)	4.8	46.9	9(8+ve)
Chickenpox	8(1.5)	2.5	25	
Bullous pemphigoid	6(1.1)	1.9	18.7	3(-ve)
Herpes labialis	2(0.4)	0.6	6.2	
Vesiculobullous lesion (not verified)	1(0.2)	0.3	3.1	
Total number of patients with any vesiculobullous disease	32 (6)	10.2	100	
***Ulcerative conditions***				
RAS	16 (2.9)	5.1	66.7	1(+ve)
Drug reaction	3 (0.6)	1.0	12.5	
Stevens-Johonson syndrome	2 (0.4)	0.6	8.3	
Erythema multiforme	1 (0.2)	0.3	4.2	
Traumatic ulcer	1 (0.2)	0.3	4.2	
Unspecified ulcer	1 (0.2)	0.3	4.2	
Total number of patients with any ulcerative condition	24 (4.5)	7.6	100	
***Pigmented Lesions***				
Melanotic macules	20 (3.7)	6.3	95	1(+ve)
Gingival tattoo	1 (0.2)	0.3	4.8	
Total number of patients with any pigmented Lesion	21 (3.9)	6.7	100	
***Connective tissue lesions***				
Fibroepithelial polyp	9 (1.7)	2.9	81.8	4(+ve)
Denture induced fibrous hyperplasia	2 (0.4)	0.6	18.2	
Total number of patients with any connective tissue lesion	11 (2.1)	3.5	100	
***Fungal infections***				
Acute erythematous candidiasis	3 (0.6)	1.0	30	1(-ve)
Median rhomboid glossitis	3 (0.6)	1.0	30	
Pseodomembranous candidiasis	3 (0.6)	1.0	30	
Angular cheilitis	2 (0.4)	0.6	20	
Chronic hyperplastic candidiasis	1 (0.2)	0.3	10	
Total number of patients with any fungal infection ¶	10 (1.8)	3.2	100	
***Benign nonodontogenic tumors***				
Soft tumor like lesion	4 (0.7)	1.3	66.7	1(-ve)
Exostosis	1 (0.2)	0.3	16.7	
Palatal tori	1 (0.2)	0.3	16.7	
Total number of patients with any benign nonodontogenic tumor	6 (11)	1.9	100	
***Perioral lesions***				
Perioral dermatitis	4 (0.7)	1.3	80	
Perioral wart	1 (0.2)	0.3	20	
Total number of patients with any perioral lesion	5 (0.9)	1.6	100	
***Lip lesions***				
Unspecified cheilitis	2 (0.4)	0.6	40	
Hypopigmented lips	2 (0.4)	0.6	40	
Cheilitis glandularis	1 (0.2)	0.3	20	
Total number of patients with any lip lesion	5 (1.0)	1.6	100	
***Verrucal papillary lesions***				
Papillary hyperplasia	3 (0.6)	1.0	75	1(+ve)
Focal epithelial hyperplasia	1 (0.2)	0.3	25	
Total number of patients with any verrucal papillary lesion	4 (0.7)	1.3	100	
***Salivary gland diseases***				
Mucocele	2 (0.4)	0.6	100	2(+ve)
Total number of patients with any salivary gland disease	2			
***Malignant tumours***				
Kaposi sarcoma	1 (0.2)	0.3	100	
Total number of patients with any malignant tumour	1			

If we exclude the diagnosis of (fissured tongue + geographic tongue) as one disease entity;

• Total no. of fissured tongue will be 38 (7% of study population)

• Total no. of geographic tongue will be 35 (6.4% of study population)

* (+ve) = Confirmed diagnosis

§ (-ve) = Not confirmed, final diagnosis based on history and clinical picture.

¶ The sum of the categories listed may not equal the total number due to the present of more than one lesion in one patient.

Table 3 depicts the frequency distribution of OML groups within each skin disease group investigated. OML occurred most frequently in the group of *skin vesiculobullous reaction pattern *(72.2%), followed in descending order by *tumours *(66.7%), and *lichenoid reaction pattern *(60.5%). OML occurred least frequently in the skin disease group of *spongiotic reaction pattern *(46.8%). *Tongue lesions *were the most frequently occurring OML group across the various skin diseases. The highest prevalence (33.3%) of the *tongue lesions *was found among *psoriasiform reaction pattern*. On the other hand, *white lesions *occurred most frequently in the skin disease groups of *disorders of pigmentation *and *lichenoid reaction pattern *amounting to 43.9% and 34.2%, respectively.

**Table 3:** Frequency distribution n (%) of patients with any OML within the 10 most common groups of skin diseased patients

Skin diseases (groups)	Patients with any OML (n = 315)	Tongue (n = 127)	White (n = 104)	Red (n = 60)	Vesiculobullous (n = 32)	Ulcerative (n = 24)	Pigmented (n = 21)	Connective (n = 11)	Fungal (n = 10)	Others (n = 23)
**Spongiotic**	59	27	22	16	-	3	6	-	1	5
**(n = 126)**	(46.8)	(21.4)	(17.5)	(12.7)		(02.4)	(04.8)		(00.8)	(04.0)
**Infectious lesions**	65	25	18	12	9	2	3	-	1	7
**(n = 115)**	(56.5)	(21.7)	(15.7)	(10.4)	(07.8)	(01.7)	(02.6)		(00.9)	(06.1)
**Vesiculobullous**	39	11	3	-	22	5	3	1	4	2
**(n = 54)**	(72.2)	(20.4)	(05.6)		(40.7)	(09.3)	(05.6)	(01.9)	(07.4)	(03.7)
**Cutaneous**	26	9	9	3	-	3	1	3	-	1
**(n = 48)**	(54.2)	(18.8)	(18.8)	(06.3)		(06.3)	(02.1)	(06.3)		(02.1)
**Pigmentation**	24	7	18	7	-	-	1	2	-	-
**(n = 41)**	(58.5)	(17.1)	(43.9)	(17.1)			(02.4)	(04.9)		
**Lichenoid**	23	11	13	5	-	2	1	1	1	
**(n = 38)**	(60.5)	(28.9)	(34.2)	(13.2)		(05.3)	(02.6)	(02.6)	(02.6)	
**Psoriasiform**	17	10	7	5	-	-	1	1	1	1
**(n = 30)**	(56.7)	(33.3)	(23.3)	(16.7)			(03.3)	(03.3)	(03.3)	(03.3)
**Vasculopathic**	13	8	2	3	-	3	1	-	1	-
**(n = 25)**	(52.0)	(32.0)	(08.0)	(12.0)		(12.0)	(04.0)		(04.0)	
**Tumour**	8	3	1	-	-	1		1	-	2
**(n = 12)**	(66.7)	(25.0)	(08.3)			(08.3)		(08.3)		(16.7)
**Others**	41	16	11	9	1	5	4	2	1	5
**(n = 55)**	(74.5)	(29.1)	(20.0)	(16.4)	(01.8)	(09.1)	(07.3)	(03.6)	(01.8)	(09.1)

• OML (others); non odontogenic tumors, perioral lesions, lip lesions, verrucal papillary lesions, mucocele, oral malignancy

• Some patients were recorded more than one time because they appeared under more than one disease group

As shown in Table 4, OML occurred more frequently in older than younger patients (62.4% versus 52.7%, p < 0.05), in males than in females (63.2% versus 52.6%, p < 0.05) and more frequently in patients with than without a medical diagnosis (65% versus 51.9%, p < 0.05). Moreover, OML occurred more frequently in toombak users than in their counterparts who had never used toombak (77.9% versus 54.8%, p < 0.00).

**Table 4:** Skin diseases with oral lesions and with at least two OML by socio-demographic and behavioural factors (n = 544)

	Skin disease with OML	Skin disease with ≥ 2 oral lesions
	N = 315	N = 113
***Age***	**n (%)**	**n (%)**
Younger (18-32 yrs)	147 (52.7)	42 (28.6)
Older (33-85 yrs)	159 (62.4)*	69 (43.4)
***Sex***		
Females	143 (52.6)	43 (30.1)
Males	172 (63.2)*	70 (40.7)
***Employment status***		
Employed	197 (60.6)	69 (35)
Non employed	117 (53.7)	44 (37.6)
***Education***		
Lower education (illiterate/primary school)	162 (60.7)	65 (40.1)
Higher education	146 (54.5)	47 (32.2)
***Residence***		
Khartoum	235 (56.6)	88 (37.4)
Outside Khartoum	77 (62.1)	24 (31.2)
***Medical diagnosis***		
No systemic condition	154 (51.9)	53 (34.4)
Presence of systemic condition	161 (65.2)*	60 (37.3)
***Toombak use***		
Never	256 (54.8)	90 (35.2)
Former/current use	53 (77.9)**	22 (41.5)
***Smoking***		
Never	251 (56.8)	89 (35.5)
Former/current use	60 (63.2)	23 (38.3)
***Alcohol***		
Never	293 (57.2)	103 (35.2)
Former/current use	16 (69.6)	9 (56.3)

• * p < 0.05 ** p < 0.00

• The sum of the categories listed may not equal the total number due to lack of information

## Discussion

### Frequency and diversity of oral mucosal lesions

To our knowledge this study is the first to assess the frequency and diversity of OML in dermatologic patients, a selected group of the Sudanese adult population. The study group comprised patients with a wide range of dermatological diseases, yielding small numbers in each group, thus limiting the probability for stratified analyses. The most frequently occurring groups of dermatological diseases were *spongiotic reaction pattern*, *infectious diseases*, and *vesiculobullous diseases*. This accords with the results of a recent survey by the International Foundation of Dermatology, reporting that infectious disease, dermatitis, and HIV-related skin disease are the main skin dermatological conditions at the community level worldwide [30].

According to the present results, about 58% of the subjects investigated suffered from at least one type of OML, and the occurrence of any OML varied across groups of dermatological diseases from 46.8% in *spongiotic *to 72.2% in *vesiculobullous reaction patterns*. *Tongue lesions *were the most frequently occurring OML group (23.3%) followed by *white lesions *(19%), *red and blue lesions *(11%) and *vesiculobullous diseases *(6%). *White lesions *and *red and blue lesions *varied systematically with age, being most frequent in older persons, whereas *ulcerative conditions *were most common in males. Coated tongue, snuff dippers lesion, erythema and pemphigus vulgaris were the OML most frequently observed in the groups of *tongue lesions*, *white lesions*, *red and blue lesions *and *vesiculobullous diseases*, respectively.

### Study Limitations

The present findings should be interpreted with caution due to some limitations. Patients' refusal to volunteer for biopsy might have led to some misclassifications. Thus, some lesions that needed histological confirmation (leukoplakia, lupus erythematosus, pemphigus vulgaris, fibroepithelial polyp, chronic hyperplastic candidiasis, cheilitis glandularis, focal epithelial hyperplasia, Kaposi's sarcoma and some others) were diagnosed clinically and might contain error. Absence of standard methodological approaches and lack of agreed-upon diagnostic criteria, make comparison of epidemiological studies concerning the prevalence of OML difficult. In spite of the limitations associated with diagnostic criteria, all mucosal pathological alterations were identified in the present study.

Being a hospital based study; it is not possible to generalize from the study group to any larger population of skin diseased individuals inside or outside Khartoum. This is due to the rich geographical and socio-cultural diversity within Sudan, as well as the low utilization rate of health facilities generally observed in any developing country [31,32]. Although the KTH received patients that have been referred from all over the country, biases in the study group might have been introduced due to differing referral procedures as well as the moderate response rate.

It is unsure how close an approximation the present figures are to the prevalence of OML in the general adult population of Sudan. Probably, the rates of OML presented in this study might be overestimated both with respect to the Sudanese population in general as well as to the population of adults suffering dermatological problems. Self-selection bias was considered to influence the result of the study as patients were more likely to respond when they had OML (the characteristic of interest). Moreover, with respect to the diversity of the types of OML, the present figures might be biased towards those for which people are more inclined to seek treatment, whereas other conditions are less likely to be identified in hospital based prevalence studies. Community based surveys based on random samples from the broader adult population should be recommended for future studies to estimate the actual prevalence and the health burden of OML in this country.

Since the precision of estimates tend to decrease with decreasing prevalence, the prevalence rates of rare conditions (≤ 1%) should be interpreted with particular caution. In addition, populations with different distributions of the risk factors identified for OML are not directly comparable without adjustment. Noteworthy the absence of an official patient's medical journal has created uncertainty regarding participants' self-reported medical condition and lifestyle patterns. A major limitation of self-reported data is recall biases in terms of underreporting of socially undesired events and a tendency to recall events as having occurred more recently than they actually did [33]. Sensitive events, tobacco and alcohol use and some medical diagnoses would probably be under reported due to social stigma and social desirability.

### Comparison of present findings with those of previous studies

In spite of its limitations, the present study provides important information about the frequency and diversity of OML in patients with various dermatological diseases as well as the social and behavioural factors that discriminate between skin diseased patients with and without OML. Moreover, OML in the present study may appear as a part of mucocutaneous diseases, a manifestation of systemic diseases (metabolic or immunological), or an expression of drug reaction. Some OML diagnosed could be attributed to trauma, infection, or denture use, or they could be a manifestation of specific cultural habits, like use of toombak. Due to the cross sectional nature of the present study, any causal relationship could, however, not be concluded upon.

Compared with the frequency of patients with OML observed in this study (57.9%), previous ones have shown point prevalence in the range 25% - 61.6% [6,9,34-36]. Specifically, the frequency of patients with OML in the present study group was higher than those observed in the Cambodian (4.9%) [37] Malaysian (9.7%) [7], Spanish (51%) [12] and Turkish (42%) populations [36]. It was lower than that observed in population in Ljubljana (61.6%), but almost similar to the prevalence estimated in Spanish dental patients (58.7%) [8,35]. In accordance with the NHANES III [6] and the Swedish study published by Axell [5], the present study used the WHO diagnostic criteria and Axell's diagnostic criteria [5,25]. Thus, the present results are to some extent comparable with those previous studies, in spite that NHANES III and the study by Axell used large probability samples from the general populations. The frequency observed in this study was higher than that reported in NHANES III, amounting 28% in US adults aged 17 years and above.

Consistent with the results of NHANES III and other studies, the frequency of patients with OML presented in this study varied systematically and positively with being a male and with increasing age. Other epidemiological studies have shown an opposite sex gradient or no systematic variation according to sex [9,38,39]. Sex differences in the occurrence of OML might be attributed to the high consumption of toombak by males, differences in genetic factors, social responsibility and masculinity believes [40]. Use of toombak was reported by 12.5% of the total study group. In a study emanating from northern Sudan, the frequency of toombak use was estimated to 40% (43, 44). Males adopt a more active outdoor life-style and are exposed to some environmental risk factors to a higher extent than women. In contrast, women are more health conscious and faster to detect abnormality in earlier stages. Older people have higher risk to develop chronic diseases in general because of increased risk with increasing age due to metabolic changes, medications, prosthetic use, and psychological problem. Moreover, economic constraints and physical status of older people may limit their access to health care services [41,42].

Epidemiological studies have revealed that *tongue lesions *constitute a considerable proportion of OML, with prevalence rates varying across different parts of the world. Number and type of *tongue lesions *involved in different studies have been an important factor in this variability. The present figure amounting to 23%, is lower than that reported in some previous studies [43,44], but higher than the rates assessed in NHANES III and in the Hungarian population [6,45]. Of interest was that 17 out of 30 patients (56.7%) with *psoriasiform reaction pattern *had OML and that *tongue lesions *(33.3%) were the most frequently occurring OML in this particular dermatological disease group (Table 3). A study of Brazilian psoriatic patients revealed that 59% presented with tongue lesions, which was the most dominant OML [46]. Similar findings have been reported by Hernandez-Perez et al [19]. With respect to fissured tongue, the total of 7% of patients with fissured tongue observed in this study corroborates the range reported previously [5,45,47,48]. Some few studies have reported high frequency of fissured tongue [35,39,43]. Over the past few years an association between geographic tongue, fissured tongue and psoriasis has been postulated. Some authors believe that it is a natural developmental anomaly and a coincidence finding [46,49] while others suggest a pathogenic relation between them [50].

Snuff dipper's lesion was observed in 5.5% of the study group (Table 2). This frequency is higher than that reported in the American and Kenyan population (1.2% and 0.4%, respectively) [6,51], but lower than that observed in the Swedish population (15.9%) [52]. Toombak has been known to play a major role in the aetiology of oral cancer in the Sudan [23]. It contains at least 100-fold higher concentrations of the carcinogenic factor tobacco specific N-nitrosamines compared with American and Swedish commercial snuff brands [53]. A recent study showed that toombak induces DNA damage and cell death in normal human oral cells more than the Swedish snuff [54].

The frequency of oral leukoplakia (3.1%) disclosed in this study is comparable to findings from Sweden (3.6%), but higher than that reported in NHANES III (0.38%) [6]. Leukoplakia is a premalignant lesion with transformation rates varying from 15.6% to 39.2% [55]. It is highly associated with cigarette smoking [8,27,56]. Although we have not done any further analysis of smoking as a possible risk factor of leukoplakia, the low frequency rate of cigarette smoking concomitant with a relatively high frequency of oral leukoplakia as observed in this study deserves further investigation. The high frequency of leukoplakia should be taken seriously as leukoplakia in non-smokers is more likely to undergo malignant transformation than leukoplakia in smokers [55].

A total of 4 patients (0.7%) with oral manifestation of discoid lupus erythematosus (DLE) on vermilion border were diagnosed in this study (Table 2). This condition has rarely been registered in OML investigation studies. Axel [5] reported 0.01% in a Swedish population, while Ramirez et al [21] reported 5% in lupus patients referred to a dermatology clinic because of oral complaints. The difference between the present figure and that reported by Ramirez et al may be attributed to the fact that although both data were collected in dermatology clinic, the selection of patients was different. The precancerous potential of oral DLE is a controversial topic. Lu and Le [57] reported an incidence of 13.6% epithelial dysplasia in DLE. Another report from Scully et al [58] postulated that DLE on the lip showed a premalignant potential. Sun exposure plays a crucial role in the induction or exacerbation of the lupus erythematosus and actinic cheilitis [28,59,60]. In connection to that, Wakisa et al [61] reported oral cell carcinoma on lips of black patients with oral DLE. Noteworthy the tropical climate in Sudan and the summer temperature which often exceed 43°C has to be considered in interpreting such lesions.

Frequency of recurrent aphthous stomatitis (RAS) has been recorded as life time prevalence, point prevalence and as combination of both. The present study revealed a point prevalence of 2.9%, which is higher than 2% and 0.8% reported by Axell [5] and NHANES III [6] respectively. Yet, it was lower than 60% and 55% in US female student nurses and professional school students respectively [62]. This illustrates how RAS varies according to the study group examined. A number of factors have been attributed to the occurrence of this pathology, including immune dysfunction [28].

## Conclusions

In conclusion, taking into consideration the selected study group and the cross-sectional design of the study, the results presented here cannot be generalized to a broader population or discussed in terms of causal relationship. The results revealed that OML were frequently diagnosed in skin diseased patients attending KTH and varied systematically with age, gender, systemic condition and use of toombak. Thus, this study provides information regarding the frequency, diversity and socio-behavioural correlates of OML of an important sub group of the Sudanese population that has never been disclosed before. Of particular significance are those lesions having a potential of malignant transformation. Accordingly, frequent and regular inspection of the oral cavity of the skin diseased patients must be emphasized. Consequently, an interdisciplinary approach in the management of such patients is highly recommended.

## Competing interests

The authors declare that they have no competing interests.

## Authors' contributions

NMS was the main author conceived and designed the study, collected data, performed statistical analysis and drafted the manuscript. ANÅ was the co-supervisor, participated and guided the study design and has been actively involved in all stages throughout the work, especially statistical analyses and epidemiological analyses of data. RWA facilitated the field work and has been providing critical comments on the study design and the manuscript. HS was the main dermatologist who examined and diagnosed all the patients. ACJ was the main supervisor, supervising and guiding the whole work and confirmed and approved all the diagnosis of oral lesions. All authors read and approved the final manuscript.

## Pre-publication history

The pre-publication history for this paper can be accessed here:

http://www.biomedcentral.com/1472-6831/11/24/prepub
